# Ambulatory Monitoring of Subglottal Pressure Estimated from Neck-Surface Vibration in Individuals with and without Voice Disorders

**DOI:** 10.3390/app122110692

**Published:** 2022-10-22

**Authors:** Juan P. Cortés, Jon Z. Lin, Katherine L. Marks, Víctor M. Espinoza, Emiro J. Ibarra, Matías Zañartu, Robert E. Hillman, Daryush D. Mehta

**Affiliations:** 1 Center for Laryngeal Surgery and Voice Rehabilitation, Massachusetts General Hospital, Boston, MA 02114, USA; 2 Department of Electronic Engineering, Universidad Técnica Federico Santa María, Valparaíso 2390123, Chile; 3 Communication Sciences and Disorders, MGH Institute of Health Professions, Boston, MA 02129, USA; 4 Speech, Language & Hearing Sciences Department, College of Health & Rehabilitation: Sargent College, Boston University, Boston, MA 02215, USA; 5 Department of Sound, Universidad de Chile, Santiago 8380453, Chile; 6 Department of Surgery, Massachusetts General Hospital–Harvard Medical School, Boston, MA 02114, USA; 7 Speech and Hearing Bioscience and Technology, Division of Medical Sciences, Harvard Medical School, Boston, MA 02115, USA

**Keywords:** subglottal pressure, clinical voice assessment, neck-surface accelerometer, ambulatory voice monitoring

## Abstract

The aerodynamic voice assessment of subglottal air pressure can discriminate between speakers with typical voices from patients with voice disorders, with further evidence validating subglottal pressure as a clinical outcome measure. Although estimating subglottal pressure during phonation is an important component of a standard voice assessment, current methods for estimating subglottal pressure rely on non-natural speech tasks in a clinical or laboratory setting. This study reports on the validation of a method for subglottal pressure estimation in individuals with and without voice disorders that can be translated to connected speech to enable the monitoring of vocal function and behavior in real-world settings. During a laboratory calibration session, a participant-specific multiple regression model was derived to estimate subglottal pressure from a neck-surface vibration signal that can be recorded during natural speech production. The model was derived for vocally typical individuals and patients diagnosed with phonotraumatic vocal fold lesions, primary muscle tension dysphonia, and unilateral vocal fold paralysis. Estimates of subglottal pressure using the developed method exhibited significantly lower error than alternative methods in the literature, with average errors ranging from 1.13 to 2.08 cm H_2_O for the participant groups. The model was then applied during activities of daily living, thus yielding ambulatory estimates of subglottal pressure for the first time in these populations. Results point to the feasibility and potential of real-time monitoring of subglottal pressure during an individual’s daily life for the prevention, assessment, and treatment of voice disorders.

## Featured Application

A non-invasive and accurate method for estimating subglottal pressure during naturalistic speech production could significantly improve the clinical assessment, treatment, and prevention of voice disorders. Ambulatory monitoring and biofeedback could thus be performed in real-world settings as speakers respond to daily vocal demands.

## 1. Introduction

In the United States, voice disorders affect approximately 30% of the adult population at some point in their lives, with about 25 million individuals suffering from a voice-related complaint at some point in their lives [1,2]. The impact of living with a voice disorder is far-reaching, often exacting significant financial, social, professional, and psychological consequences [3]. The societal burden of voice disorders has been estimated to reach up to USD 13.5 billion dollars each year due to work-related disability, lost productivity, and healthcare costs [3–5]. Individuals with voice disorders often suffer from heightened sensations of vocal effort and fatigue while speaking, which are typically attributed to inefficient vocal function and behavior [6–8]. Thus, there is a strong clinical motivation for the objective measurement of acoustic and aerodynamic parameters related to vocal efficiency that can provide a window into the daily life of these individuals.

Subglottal air pressure (Ps) during voice production has been linked with the self-perception of vocal effort [9–11] and is an important part of objective measures of vocal efficiency [12–16]. A positive aerodynamic pressure gradient across the glottis facilitates self-sustained oscillation of the vocal folds. This oscillation modulates the laryngeal airflow from the lungs and provides energy excitation to the vocal tract to output what we measure and perceive auditorily as the acoustic voice signal. Ps plays an important part in vocal function and aids in controlling onset, offset, intensity, and fundamental frequency (*f*_o_) [17–20]. Measures of Ps and measures derived from Ps and laryngeal airflow measures (such as laryngeal resistance and vocal efficiency measures) can discriminate patients with voice disorders from individuals with typical voices and discriminate vocal characteristics before and after the clinical management of a voice disorder [21–28]. The efficiency with which aerodynamic power is transferred into acoustic power can be an indicator of vocal health [29].

### 1.1. Traditional Methods of Subglottal Pressure Estimation

The direct measurement of Ps can be accomplished but is rarely performed due to its invasive nature, including tracheal puncturing for subglottal sensor positioning [30,31] or transglottal placement of pressure transducers [32,33]. Traditionally, indirect methods of Ps estimation were cumbersome and included full-body plethysmography (measuring the pressure changes outside the body in a closed-loop environment) [34,35] and an esophageal balloon technique (measuring the pressure against the esophageal wall) [32,36]. More routinely in current practice, indirect estimation of Ps involves the production of sustained phonation at a given pitch and loudness that is interrupted volitionally by a bilabial, unvoiced consonant (e.g., /p/) [37,38]. Using this method, the subglottal pressure is inferred from the intraoral pressure measured during the consonant when Ps equilibrates with the intraoral pressure. The latter is measured using a pressure sensor attached to a flexible tube inserted between the lips, which form a seal around the tubing during the consonant production. A non-volitional airflow interruption technique has been developed using a mechanical system but requires additional specialized hardware and can suffer from triggering undesirable involuntary laryngeal reactions [39,40]. Even though Ps estimates have provided valuable information about vocal function and is a standard aerodynamic measurement in the clinic [41], their information has been inherently limited to sustained vowel contexts. Thus, there is a strong desire to develop a method to estimate Ps during natural speech production where loudness, pitch, and voice quality can vary dynamically, especially in the context of real-world environments and situations where individuals experience their vocal symptoms.

### 1.2. Subglottal Pressure Estimation from Anterior Neck-Surface Vibration

Recent lines of research have focused on estimating Ps from anterior neck-surface vibration using a miniature accelerometer (ACC) placed below the level of the glottis [42–46]. This ACC sensor is a piezo-ceramic vibration transducer that measures the second derivative (acceleration) of the one-dimensional displacement perpendicular to the surface of the neck skin. Monitoring vocal characteristics using ACC sensors is desirable because these sensors have been shown to be robust to airborne acoustic noise relative to contact microphones [47–49], produce a voice-related signal that is not filtered by vocal tract resonances and thus unintelligible (maintaining confidentiality) [50], and can be part of wearable systems for long-term ambulatory voice monitoring [51–53]. Positioning the ACC sensor below the glottis enables measurement of Ps-related information due to coupling of aerodynamic pressures in the trachea through the tracheal and neck tissue to the surface of the skin [54,55]. Amplitude and frequency properties of the subglottal ACC signal have been shown to correlate highly with properties of the associated acoustic voice signal, including *f*_o_ and variability metrics such as jitter and cepstral peak prominence (CPP) [56]. In fact, the root-mean-square (RMS) value of the ACC signal has been used as the primary correlate of acoustic sound pressure level (SPL) through simple linear mapping [57]; when the phonatory SPL increases, the RMS magnitude of the ACC signal generally increases as well. This mapping approximately holds across loudness and pitch contexts and can be used as a calibration step so that the SPL and derived vocal dose measures can be derived from the ACC signal in ambulatory contexts [58,59].

ACC-derived measures of SPL and *f*_o_ can then be input into an empirical formula found in the literature to estimate Ps [58,60,61]. Using this approach, the derivation of ACC-based Ps is applied on a person-specific basis since the RMS-based mapping to SPL is not universal and depends on the variability in neck tissue morphology and acoustic-aerodynamic relations across individuals [57]. The accuracy of estimating Ps in this manner is thus dependent on the validity of the model, as well as the accuracy in estimating SPL and *f*_o_. The accuracy in estimating *f*_o_ from the ACC signal is very high [56], validating why ACC signals have been used for noise-robust *f*_o_ tracking for decades [49]. However, the accuracy in estimating SPL from the ACC signal is lower, with average confidence intervals lying within ±6 dB [57], which is a range spanning soft-to-loud loudness levels [62]. ACC-based estimation of SPL can also be affected by other factors such as vocal tract shape (vowel type) and glottal configuration (leading to different voice qualities). For example, evidence from vocally typical speakers points to higher correlations between ACC RMS and Ps than between ACC RMS and SPL when investigating the impact of variations in vowel type and pitch [42]. Thus, this alternative approach to ACC-based Ps estimation bypasses the need for SPL and *f*_o_ estimation, with the RMS value of the ACC signal acting as a person-specific correlate of Ps in modal phonation.

The effects of non-modal phonation (breathiness, roughness, and strain) on the linear ACC RMS–Ps mapping were subsequently studied in vocally typical speakers [63]. Results demonstrated, as expected, a statistically significant linear relationship between ACC RMS and Ps for each speaker producing modal phonation; however, the linear model exhibited larger intercepts when non-modal phonatory conditions were elicited (slopes were less affected by non-modal phonation). In a follow-up study of patients with voice disorders, patients exhibited higher model intercepts; i.e., higher levels of Ps given similar ACC RMS values when compared with vocally typical individuals [64]. In particular, the intercepts of the regression line were greater, on average, for non-modal phonatory conditions relative to modal phonation. The Ps required for speakers to initiate and maintain voicing tended to be higher for the same neck-surface vibration amplitude when phonation was breathy, rough, or strained. The conclusion of these studies was that the baseline regression line between ACC RMS and Ps can be significantly affected by the presence of non-modal phonatory characteristics [64] or phonation associated with increased vocal effort [43].

Two additional Ps estimation approaches have been proposed to account for the effects of non-modal and disordered phonation. Both approaches rely on the computation of additional features from the ACC signal that are theoretically and empirically linked to non-modal and disordered phonatory function. These features include global vocal function measures, such as CPP [56,65–69], and glottal airflow measures, such as peak-to-peak airflow, open quotient, maximum flow declination rate, and spectral tilt [28,50,70]. In the first approach, these ACC-based features are input into a person-specific multiple linear regression model that is trained using phonation from each speaker at different vocal intensity levels [44]. In the second approach, the ACC-based features are input into a nonlinear neural network model that is trained using thousands of synthesized vowels generated by a computational voice production model sweeping across thousands of combinations of control parameters [46]. The accuracy of these two approaches was only reported for phonation by vocally typical speakers. The current study extends on this past work by assessing the performance of multiple ACC-based methods for Ps estimation in patients with voice disorders.

### 1.3. Clinical Motivation for Ambulatory Monitoring of Subglottal Pressure

There is strong evidence that laboratory measures of Ps can discriminate patients with vocal hyperfunction from vocally typical control speakers, with effect sizes that appear to be even higher than other aerodynamic measures related to glottal airflow characteristics [70]. In addition, patients with phonotraumatic vocal fold lesions (nodules or polyps) have been reported to exhibit Ps values over two standard deviations greater than normative Ps values [28]. Changes in Ps have also been associated with the post-surgical outcomes in patients with UVFP [71] and laryngeal cancer [24]. However, the literature has relied solely upon estimating Ps during non-natural syllable strings when studying the effects of voice disorders on Ps. Furthermore, the studies have assessed vocal behavior in controlled laboratory or clinical settings that provide only brief snapshots of vocal function [23,72,73]. The current study builds upon ongoing work that is advancing ACC-based technology to enable effective strategies for ambulatory voice monitoring and biofeedback [51,53,65,74–82]. Previous studies of Ps for clinical voice assessment have documented the importance of evaluating Ps in the context of the vocal SPL produced [28,62,83–86]. The current study focuses on the validity and feasibility of ambulatory Ps estimation that could then be augmented in the future with ambulatory measures of vocal SPL, as well as with perceptual ratings of vocal symptoms such as vocal effort, discomfort, and fatigue [6,87–90].

### 1.4. Study Goals

The goals of the current study are to (1) compare the predictive performance of ACC-based Ps estimation using four approaches [44,46,58] and (2) demonstrate the feasibility of the ambulatory estimation of Ps in individuals with and without voice disorders. The predictive performance of ACC-based Ps estimation was studied in the laboratory, where the reference measures of Ps were derived using the standard indirect method [41] that was modified to elicit many tokens across vocal intensity levels [13]. The infield estimation of Ps was carried out using a smartphone-based voice monitoring system [53,77] that recorded the ACC signal during one day for each study participant.

## 2. Materials and Methods

### 2.1. Study Participants

Thirty patients with voice disorders were enrolled in the study and described previously [64]: 10 with phonotraumatic vocal hyperfunction (PVH; diagnosed with nodules and/or polyps), 10 with nonphonotraumatic vocal hyperfunction (NPVH; diagnosed with primary muscle tension dysphonia), and 10 with unilateral vocal fold paralysis (UVFP). These three voice disorders were studied because of the high incidence of vocal effort complaints in these clinical populations [88], hallmarks of degraded voice quality (breathiness, hoarseness, and/or strain) that could affect ACC-based Ps estimation, and a previous laboratory study of Ps in these patient cohorts [64]. Diagnoses were made by a laryngologist and speech-language pathologist specializing in voice disorders using a comprehensive assessment protocol that included (1) medical history information, (2) laryngeal stroboscopic imaging [91], (3) self-rated Voice-Related Quality of Life (V-RQOL) questionnaire [92], (4) clinician-rated Consensus Auditory-Perceptual Evaluation of Voice (CAPE-V) [93], and (5) objective aerodynamic and acoustic measurements of vocal function [41]. Exclusion criteria included previous voice treatment, except for one patient with UVFP, who was enrolled six weeks after an initial laryngeal medialization, and a second patient with UVFP, who was enrolled two years after an initial laryngeal medialization (glottal insufficiency persisted in these patients during the study). Data from 26 participants with typical voices from previous studies [44,63] acted as a control group, with typical sounding voices and vocal folds with straight edges exhibiting typical vibration, as assessed by a voice-specialized speech-language pathologist. Table 1 reports demographics of the patient and control groups.

### 2.2. Laboratory and Ambulatory Data Collection

**Figure 1.**
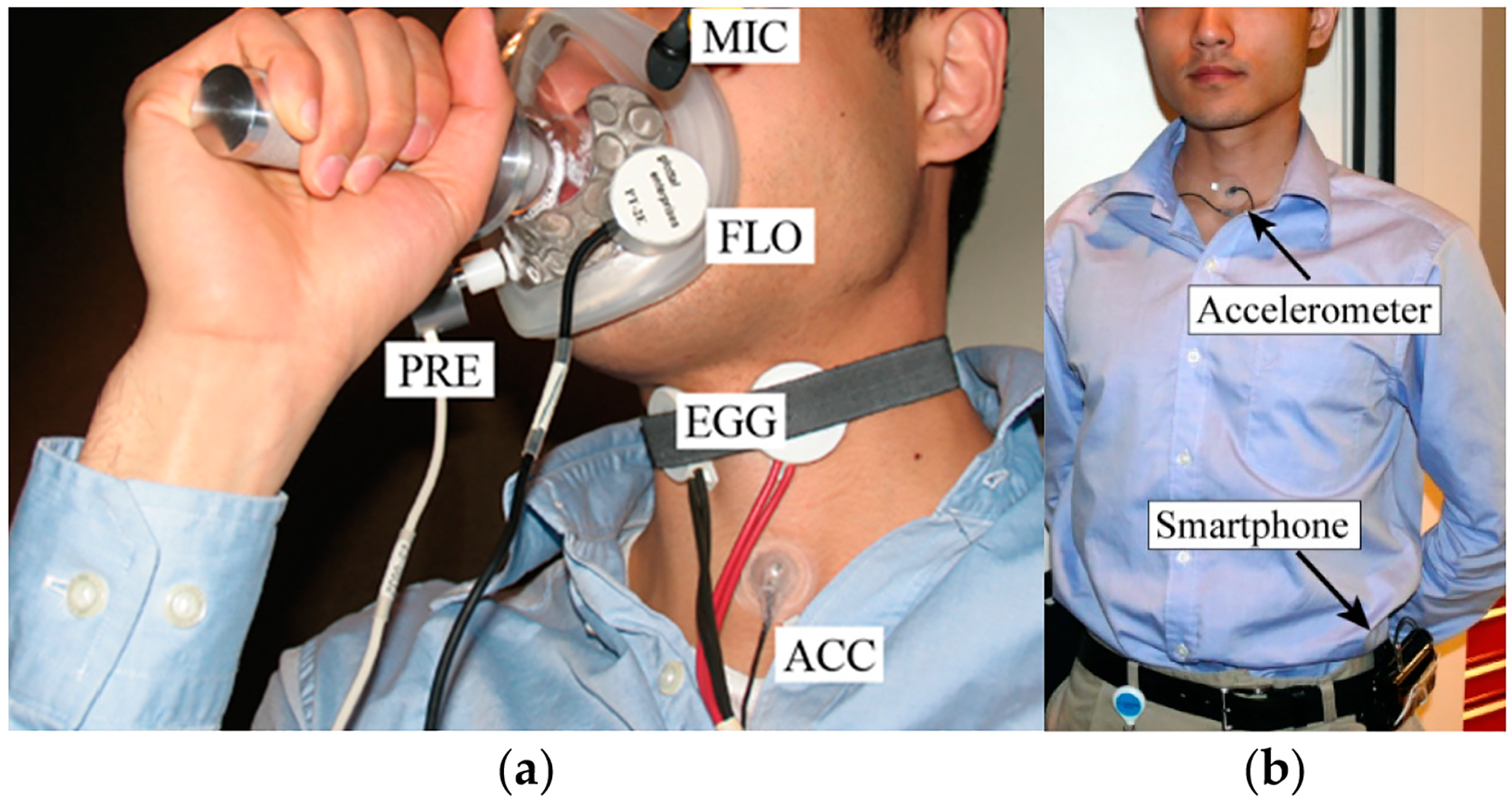
Data acquisition setups for (**a**) laboratory recordings of acoustic microphone (MIC), electroglottography (EGG), accelerometer (ACC), high-bandwidth oral airflow (FLO), and intraoral pressure (PRE); and (**b**) infield recording of the accelerometer sensor connected to a smartphone either placed in a belt holster or in a pocket. Reprinted with permission from Ref. [94]. ©2013, IEEE.

Figure 1a illustrates the laboratory setup in a sound-treated booth. The acoustic signal was recorded with a head-mounted condenser microphone positioned 15 cm from the lips (ME102, Sennheiser Electronic GmbH, Wennebostel, Germany). The laryngeal impedance signal was recorded using an electroglottograph (EG-2, Glottal Enterprises). The oral airflow and intraoral pressure signals were recorded using an aerodynamic assessment system that consisted of a pneumotachograph mask (Glottal Enterprises, Syracuse, NY, USA) and oral airflow (PT-2E, Glottal Enterprises) and intraoral pressure (PT-75, Glottal Enterprises) sensors. These signals were sampled at 20 kHz and 16-bit quantization (Digi-data 1440A, Axon Instruments) following an analog antialiasing, lowpass filter stage with an 8 kHz cutoff frequency (CyberAmp Model 380, Axon Instruments, Union City, CA, USA). The neck-surface vibration signal was recorded using a miniature ACC sensor (BU-27135; Knowles Corp., Itasca, IL, USA) placed halfway between the thyroid prominence and the suprasternal notch using hypoallergenic double-sided tape (Model 2181, 3M, Maplewood, MN, USA). The ACC signal was sampled at 11,025 Hz and 16-bit quantization using an Android smartphone [53]. As described in prior work with the same study participants [42,44,63], each participant was asked to produced repeated /p/-vowel syllable strings from loud to soft in three vowel contexts (/pa/, /pi/, /pu/) and three pitch conditions (comfortable, higher than comfortable, and lower than comfortable). In this manner, up to 20 vowel segments could be produced in one breath; at least two trials for each vowel-pitch condition were elicited.

Figure 1b shows the ambulatory setup. Each study participant wore the smartphone-based ambulatory voice monitor [53] for one waking day. The ACC signal was calibrated for SPL in the beginning of the day using a microphone (H1 Handy Recorder, Zoom Corporation, Tokyo, Japan) held 15 cm from the lips. Smartphone prompts instructed participants to produce /a/ vowels from loud-to-soft loudness levels. Study participants carried the smartphone in their pocket or a belt holster while they went about their activities. The smartphone application required minimal user interaction during the day with only periodic system checks activated to verify that the ACC sensor was working. Participants were instructed to pause recording of the ACC signal and remove the sensor during high-intensity exercise, swimming, or showering. After the daylong recording was complete, participants brought the voice monitoring system back to research staff to download the raw ACC signal and associated log files that included applications settings and timestamped smartphone events.

### 2.3. Laboratory Data Analysis

#### 2.3.1. Signal Pre-Processing

**Figure 2.**
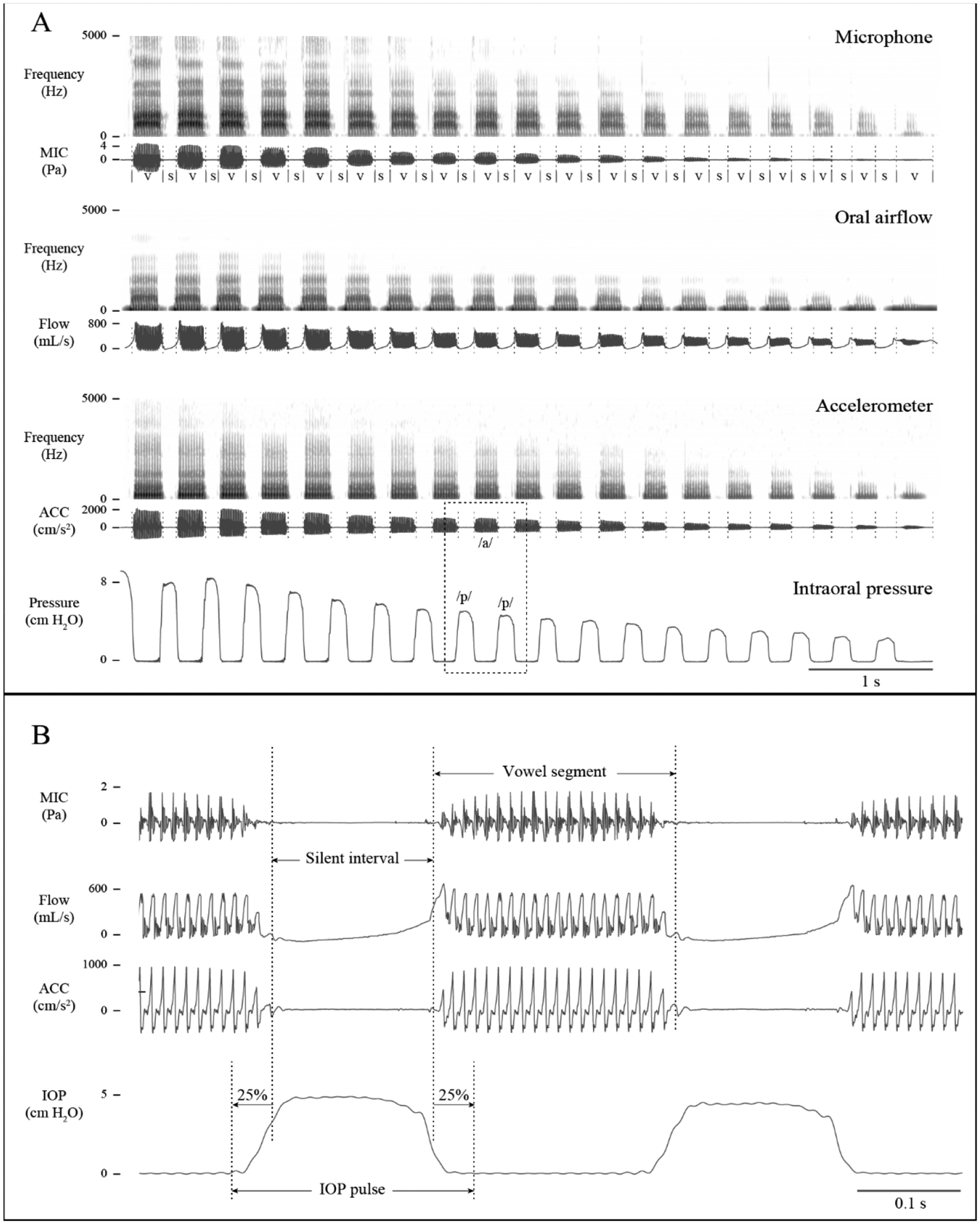
Illustration of how reference estimates of subglottal pressure were defined in a male study participant with a typical voice (M01) in modal phonation. (**A**) Time-aligned signals and associated spectrograms are plotted for the acoustic microphone (MIC), oral airflow, neck-surface accelerometer (ACC), and intraoral pressure (IOP) sensors (S = silence; V = vowel). A zoomed-in version of the boxed region is displayed in (**B**) to illustrate the definition of each vowel segment, silent interval, and IOP pulse.

Figure 2 shows example waveforms and spectrograms of oral airflow, intraoral pressure, acoustic microphone, and accelerometer signals, which were calibrated to units of milliliters per second (mL/s), centimeters of water (cm H_2_O), pascals (Pa), and vibration acceleration (cm/s^2^), respectively. Slope and intercept calibration terms were applied to each uncalibrated voltage signal. For oral airflow, a line was drawn through three points with known airflow volume velocity as output by an airflow calibration unit (Model MCU-4; Glottal Enterprises): 500 mL/s outward flow, zero flow, and 500 mL/s inward flow. For intraoral pressure, a line was drawn through five points with known pressure produced by advancing a syringe through a closed-loop system: 0, 5, 10, 15, and 20 cm H_2_O, as measured by a calibrated pressure gauge (Model PC-1; Glottal Enterprises). For the acoustic microphone signal, a line was drawn through multiple points with measured RMS levels in Pa (Model NL-20; RION Corporation, Tokyo, Japan) produced by a synthesized harmonic complex at multiple intensity levels. Finally, each ACC sensor was calibrated in units of cm/s^2^ by applying a chirp signal with known amplitude and 10–5000 Hz bandwidth using an electrodynamic vibration exciter (Mini-Shaker Type 4810, Brüel & Kjær) and a reference accelerometer (Model 4533-B, Brüel & Kjær, Nærum, Denmark) placed on a vibration isolation table (BT-2024, Newport Corp., Irvine, CA, USA). The ACC signal (up-sampled to 20 kHz) was aligned with the other recorded signals in the laboratory by maximizing the cross-correlation between the ACC signal and the microphone signal.

As described in previous work [44], vowel segments were defined by processing the microphone signal using Praat version 6.0.30 [95]. Figure 2A illustrates an example segmentation of the vowel and silent segments. Figure 2B displays a zoomed-in version of the signals with boundaries defined for each intraoral pressure plateau between the vowel segments. Reference estimates of Ps were computed for each vowel segment by the average of the peak amplitudes of the intraoral pressure plateaus preceding and following each vowel segment.

#### 2.3.2. Ps Estimation Method 1: Empirical Relationship with SPL and f_o_

The first method of Ps estimation relies on an empirical relationship found with SPL and *f*_o_. For laboratory data analysis, the SPL was computed directly from a given vowel segment in the acoustic microphone signal as

(1) $$\text{SPL}\left[\text{dB SPL}@15\text{ cm}\right]=20\;{\text{log}}_{10}\left(\frac{{\text{MIC}}_{\text{rms}}}{20\;\mu \text{Pa}}\right),$$

where MICrms is the RMS value of the middle 50 ms of the microphone vowel segment. The *f*_o_ of this 50 ms segment was computed from the accelerometer signal as the reciprocal of the first peak location in the normalized autocorrelation function; if a subharmonic exists that is at least 0.25 of the first peak, the *f*_o_ is recomputed according to the location of the subharmonic. This recomputation is necessary due to the effect of the subglottal resonance that can boost the second harmonic magnitude above that of the first harmonic. These measures of SPL and *f*_o_ were then input into the following formula to estimate Ps [58,60,61]:

(2) $$\text{Ps}\left[\text{kPa}\right]=0.14+0.06{\left(f_{\text{o}}/f_{\text{oN}}\right)}^{2}+10^{\left(\text{SPL}-88.5\right)/27.3},$$

where *f*_oN_ is the nominal speaking *f*_o_ value for males (120 Hz) and females (190 Hz).

#### 2.3.3. Ps Estimation Method 2: Linear Regression Model Using ACC Signal Magnitude Only

The second method of Ps estimation takes advantage of the strong correlation between Ps and the RMS magnitude of the ACC signal that is largely robust to vowel type and *f*_o_ when modal phonation is produced; this correlation decreases substantially when the RMS magnitude of the microphone signal is applied [42,63]. The Ps for this method is thus computed on a person-specific basis as

(3) $$\text{Ps}\left[{\text{cm H}}_{2}\text{O}\right]=\text{slope}\times \text{ACCrms}+\text{intercept},$$

where ACCrms is the root-mean-square of the middle 50 ms of the ACC vowel segment, slope is the slope of the best-fit regression line between the reference Ps estimates (in units of cm H_2_O) and ACCrms, and intercept is the intercept of the regression line.

#### 2.3.4. Ps Estimation Method 3: Multiple Linear Regression Model

The third method of Ps estimation expands the simple linear regression model in Method 2 to incorporate multiple voice production measures. The multiple linear regression model was designed to take into account non-modal phonatory effects, which prior work demonstrated increases the accuracy for estimating Ps in individuals with a typical voice [44]. The current study extends on that work by investigating whether the multiple linear regression model increases Ps estimation accuracy in patients with voice disorders as well.

The ACC-based glottal airflow waveforms were obtained using subglottal impedance-based inverse filtering (IBIF), which was applied to each vowel segment [50]. The average level of the oral airflow signal was subtracted since the ACC signal was a zeromean (AC) signal. Five IBIF model parameters were estimated for each subject: skin inertance, skin resistance, skin stiffness, tracheal length, and accelerometer position. IBIF model properties were obtained using particle swarm optimization [50,96]. For laboratory data analysis, IBIF model estimation was optimized for each vowel segment. Vowel segments with IBIF measures that were outside the physiologically relevant ranges were not included in the multiple regression (ACFL < 1 mL/s, MFDR < 1 L/s^2^, and OQ outside of 0–100% range); in addition, vowels with *f*_o_ > 500 Hz due to the known limitation of glottal inverse filtering at high values of *f*_o_.

**Figure 3.**
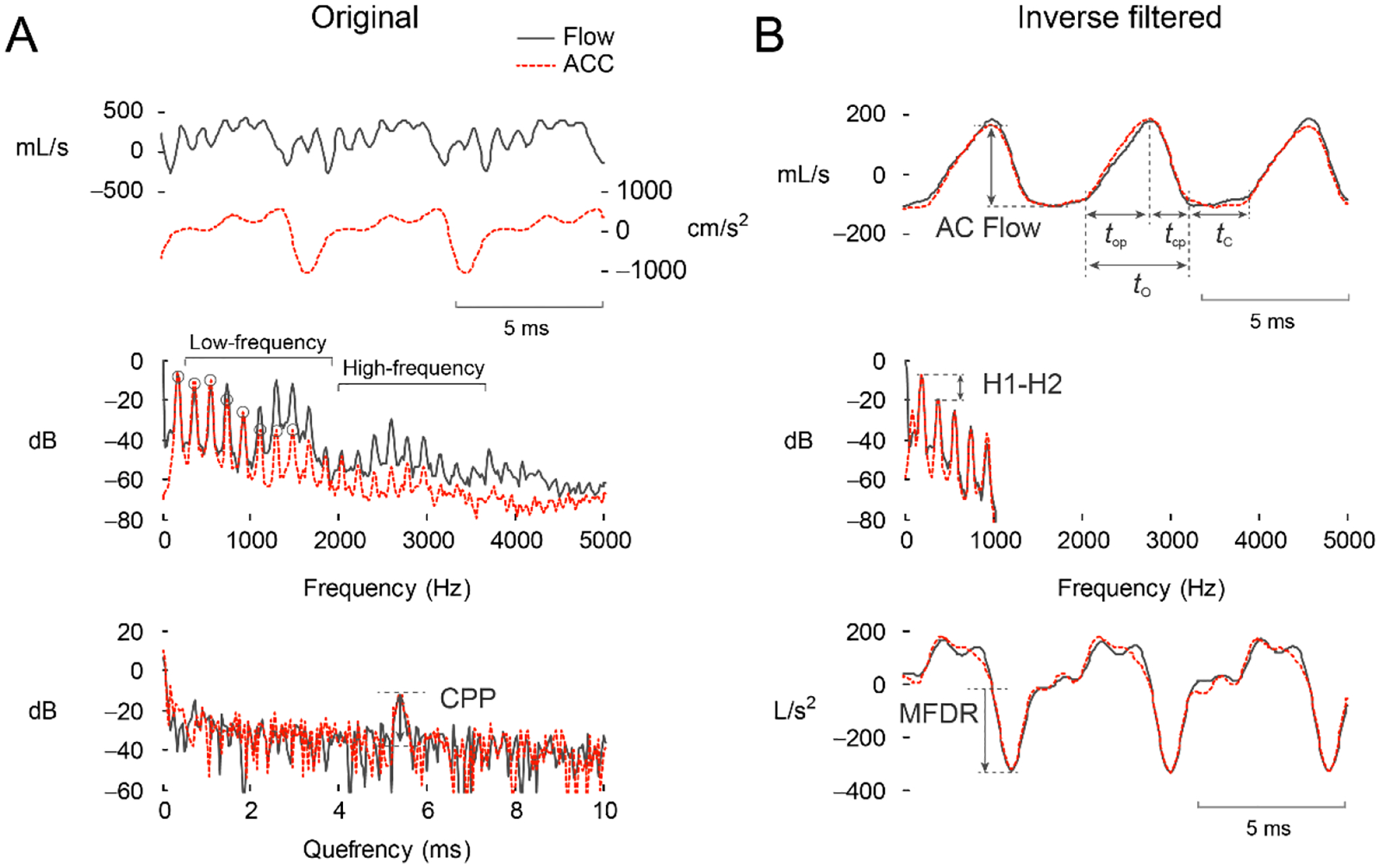
Feature extraction completed for the (**A**) originally recorded signals and (**B**) inverse-filtered versions of the oral airflow waveform (solid black) and neck-surface vibration acceleration (ACC, red-dashed). From [77].

Table 2 lists the ten ACC-based vocal function measures input into the multiple linear regression model. This set of measures was computed from each vowel segment (including all vowel types and pitch conditions) to minimize the error in predicting Ps from the ACC signal given the presence and degree of different vocal modes and pathological glottal conditions. Figure 3 illustrates the parameterization of the original ACC and inverse-filtered signal to yield the set of ten vocal function measures. The first three measures (RMS, *f*_o_, and CPP [56]) are computed directly from the raw ACC signal (Figure 3A). The rest of the seven measures are computed from the glottal airflow waveform (Figure 3B): AC flow amplitude (ACFL), maximum flow declination rate (MFDR), open quotient (OQ), speed quotient (SQ), spectral tilt (H1–H2), harmonic richness factor (HRF), and normalized amplitude quotient (NAQ).

The ten measures were input as dependent variables into a stepwise linear regression model with the reference Ps value per vowel segment as the independent variable. The stepwise regression model was described in detail in prior work [44]. Briefly, a screening step was included to determine whether each measure was sufficiently useful for inclusion into the regression model; the *p*-value of an F-statistic was computed to screen whether the additional measure contributed significantly to model prediction. The regression model was evaluated per study participant using five-fold cross-validation; i.e., training sets comprised 80% of the vowel segments and test sets comprised 20% of the remaining vowel segments (no overlap). The fold exhibiting the lowest root-mean-square error (RMSE) for the test set was selected for comparison with the other Ps estimation methods.

#### 2.3.5. Ps Estimation Method 4: Nonlinear Neural Network Model

Recently, a method was developed to combine the vocal function measures in a nonlinear neural network model in an effort to increase the accuracy of Ps estimation [46]. The neural network consisted of two fully connected hidden layers with four neurons in each layer. The input to the network included all the measures listed in Table 2, except for RMS, CPP, HRF, and NAQ. Moreover, the model included as input the acoustic SPL extracted from the microphone signal since the microphone signal is available in the laboratory setting. The number of layers and neurons was chosen according to the best results reported for laboratory test data, which are the same conditions that were analyzed in this study. The output of the network has four neurons that yield estimates of Ps, vocal fold collision pressure, and muscle activation levels of the thyroarytenoid and cricothyroid muscles. In contrast with the multiple regression model (Ps Estimation Method 3), the neural network model was pre-trained using simulated vowel signals, with radiated acoustic pressure (15 cm from the lips) ranging from 60 to 100 dB SPL, that were synthesized using a voice-production model consisting of a triangular body-cover model of the vocal folds and planar sound-wave propagation [46,97]. The multidimensional space of the model-control parameters was sampled to synthesize 13,000 vowel segments that represented a range of typical (non-disordered) phonatory configurations. The network architecture was selected to maximize the model’s predictive performance against experimental recordings of intraoral pressure in 79 vocally typical female participants uttering consecutive /pæ/ syllable strings at comfortable, loud, and soft levels, and was adjusted for the SPL conditions in this study (15 cm versus 10 cm distance microphone distance).

### 2.4. Statistical Comparison of Ps Estimation Methods

RMSE was computed as the statistical metric of accuracy when evaluating each Ps estimation method for each study participant. RMSE was computed across all vowel segments produced by a given study participant for Estimation Methods 1, 2, and 4. For Estimation Method 3, since the RMSE was computed for each of the five cross-validation test sets, the test set with the lowest RMSE was selected for comparison. A two-way analysis of variance (ANOVA) was conducted to determine any main effects of voice disorder type (phonotraumatic, non-phonotraumatic, and unilateral vocal fold paralysis), Ps estimation method (Estimation Methods 1–4), and their interaction. Post-hoc paired-samples t-tests were conducted for statistically significant interactions. Any main effects were quantified by paired Cohen’s *d* effect sizes, in particular to document the performance gain of the Ps estimation method with the lowest estimation accuracy.

### 2.5. Ambulatory Data Analysis

Initial pre-processing of the accelerometer signal was required to perform voice-activity detection using previously established methods that sought to capture phonation during daily activities and avoid non-phonatory signal artifacts (e.g., tapping, clothing rubbing on sensor, non-phonatory vibrations, and electrical noise) [77]. Table 3 lists the five features and voicing criteria needed for voice-activity detection. All features were computed over 50-ms, nonoverlapping frames. If all five features were within their respective voicing range criteria, the frame was considered voiced; otherwise, the frame was considered unvoiced. For each voiced frame, we computed the set of ten vocal function measures described in Table 2 for each study participant’s day of voice monitoring.

Since direct measurements of acoustic SPL were not available from the ambulatory voice monitor, estimates of SPL were derived from a mapping between the accelerometer and microphone recordings of an /ah/ vowel of decreasing loudness at the beginning of each participant’s monitored day [53]. Linear regression parameters were computed in a log–log space between measures of accelerometer RMS and acoustic SPL, as specified in previous studies [57]. In this manner, participant-specific slope and intercept parameters were saved and applied to the ambulatory accelerometer signal (when the microphone was not present) to map the accelerometer level (dB re 1 cm/s^2^) to units of dB SPL @ 15 cm.

As with the laboratory accelerometer data, the ambulatory accelerometer signals were calibrated to physical units of vibration acceleration (cm/s^2^) using the respective sensor’s derived calibration factor. This calibration allowed for the application of subglottal impedance-based inverse filtering to derive an estimate of the (zero-mean) glottal airflow waveform for each voiced frame. In contrast with the laboratory data analysis where oral airflow recordings were available, the ambulatory ACC signal needed to be processed using a single optimized IBIF inverse filter that was considered time-invariant and specific to each study participant to account for skin properties, tracheal geometry, and ACC sensor placement. The IBIF model was selected from a laboratory vowel /a/ segment with the highest subglottal pressure in the comfortable pitch condition (and modal voice quality for the vocally typical group). The assumption of IBIF model time-invariance was based on the model properties, which were assumed to be stable over time [50]. Even though there is some evidence that some of the neck-skin properties might change for different articulatory configurations (e.g., glottal flow estimation from an /a/ vowel compared to an /i/ vowel [99], the extent of the effect is not significant for ambulatory purposes [100].

Thus, glottal airflow features were able to be estimated in the ambulatory setting as in prior work [96]; in the current study, these features were used to aid in accurate estimation of Ps. As with the laboratory data analysis, voiced frames with IBIF measures that were outside physiologically relevant ranges were not included for Ps estimation (ACFL < 1 mL/s, MFDR < 1 L/s^2^, OQ outside of 0–100% range, and *f*_o_ > 500 Hz). In this paper, estimates of ambulatory Ps are reported using Ps Estimation Method 3, which was found to yield the lowest error among the four methods compared according to the laboratory results. The regression model of Estimation Method 3 was selected from the participant-specific laboratory training data that yielded the lowest test-set RMSE.

## 3. Results

### 3.1. Laboratory Results: Accuracy of Subglottal Pressure Estimation Using Four Methods

Table 4 lists the mean and standard deviation of the RMSE within each participant group for each of the four Ps estimation methods relative to the reference intraoral pressure in the laboratory signals during bilabial closure between sustained vowels. The auditory-perceptual rating of the overall severity of the dysphonia is also reported for the patient groups as an indicator of severity of the voice disorder and whether this severity had an effect on the Ps estimation accuracy. See Appendix A for RMSE values for each study participant. Table 5 reports the results of the ANOVA analysis, revealing statistically significant main effects of the Ps estimation method and participant group. For the main effect of method, post-hoc independent-samples t-tests revealed that Estimation Methods 1 and 4 exhibited the highest error in estimating Ps, with an overall mean (standard deviation) RMSE of 3.62 (2.08) and 3.40 (1.78) cm H_2_O, respectively (no statistical difference, *p* = 0.548). Estimation Method 1 yielded outlier values for Ps for two vocally typical participants (Ps values greater than 75 H_2_O); these values were removed prior to computing RMSE. Lower errors were exhibited by Estimation Methods 2 and 3, which were based on participant-specific models and calibration with intraoral pressure. Estimation Method 2—the single regression model based only on ACC RMS—exhibited a statistically lower error than Estimation Method 1, with an overall RMSE of 1.81 (0.76) cm H_2_O (*d* = −1.15). Estimation Method 3—the multiple regression model incorporating the complete set of vocal function measures—exhibited the lowest overall RMSE of 1.44 (0.66) cm H_2_O, a further reduction in error relative to that of Estimation Method 2 (*d* = −0.53). Within each participant group, the mean (SD) RMSE for the PVH, NPVH, UVFP, and Control groups were, respectively, 2.74 (2.03), 2.79 (1.66), 3.36 (2.32), and 2.12 (1.19) cm H_2_O. For the main effect of participant group, post-hoc independent-samples t-tests revealed that the only statistically significant difference was between RMSE for the UVFP group and Control group (d = −0.69).

### 3.2. Laboratory Results: Inclusion Frequency of Vocal Function Measures into Ps Estimation Method 3

Table 6 reports the inclusion frequency of each vocal function measure selected for prediction of Ps. This inclusion frequency table reflects how often a particular measure is included in the multiple regression model of Ps Estimation Method 3 across study participants. As expected, the RMS value of the ACC signal was included for almost all study participants, with *f*_o_, CPP, and MFDR the next most frequent measures used. OQ, NAQ, HRF, and H1–H2 were included in the regression model the least often. SQ was screened out of all the models and thus did not contribute to Ps estimation for any study participant.

### 3.3. Ambulatory Results: Feasability of Subglottal Pressure Estimation during Daily Life

**Figure 4.**
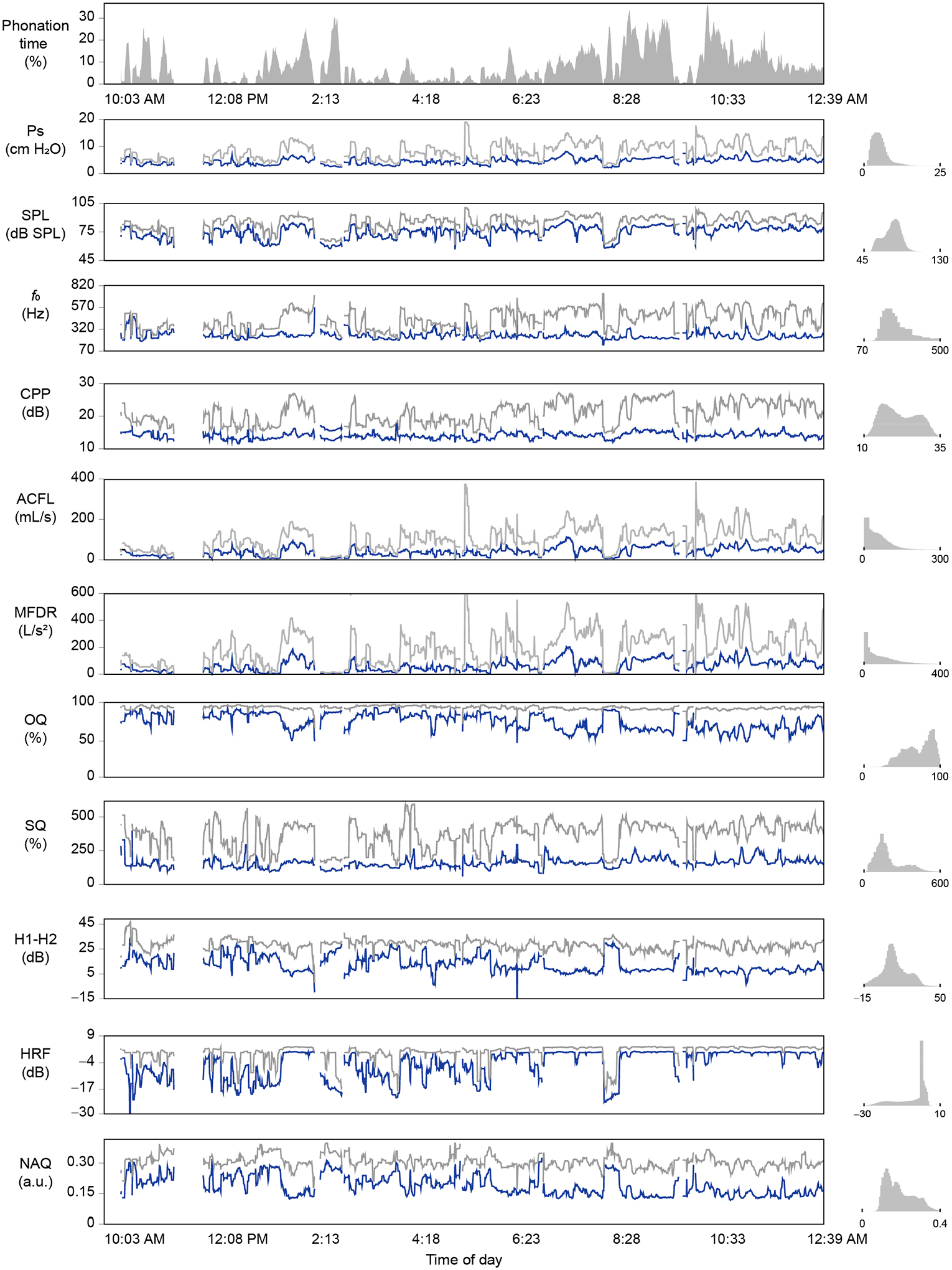
Illustration of the time-varying nature of daylong vocal function. The first plot shows the percent phonation computed over 5-min windows at intervals of half a minute. Subsequent plots are the 5-min moving averages of the median (blue line) and the 95th percentile (grey line) of the vocal function measure. Daylong histograms of each measure are shown to the right of each respective time series.

Since the lowest Ps estimation error was exhibited by Estimation Method 3 (multiple regression model), ambulatory estimates of Ps were computed using Ps Estimation Method 3 for each study participant’s monitored day. For each participant, the multiple regression model (out of the five tested in the cross-validation) that exhibited the lowest RMSE was selected to be applied to 50-ms voiced frames in the ambulatory data signal. Figure 4 displays an example analysis of the daylong voice-use profile of participant CF3, showing the time-varying contours of each vocal function measure, with the feasibility of ambulatory Ps estimation being reported for the first time in this study.

Table 7 reports the daylong summary statistics of the central tendency, dispersion, minimum, and maximum for the subglottal pressure and typically computed ambulatory vocal function and behavior (phonation time, SPL, CPP, and H1–H2). These ambulatory metrics have been studied in the pathophysiology and treatment of phonotraumatic and non-phonotraumatic vocal hyperfunction [101–105]. Summary statistics of the Ps estimates (Ps Estimation Method 3 reported) are now available to be added to the set of ambulatory vocal function measures as a key indicator of aerodynamic voice assessment. Ambulatory Ps values did not approximate statistically normal distributions; thus, the statistical mode was also reported for Ps, which resulted in values of 9.2, 8.1, 5.8, and 6.1 cm H_2_O for the participants with PVH, NPVH, UVFP, and typical voices. Since ambulatory estimates of glottal airflow features were input into the Ps estimation method, Table 8 documents the ambulatory statistics of these features for each study participant group.

**Figure 5.**
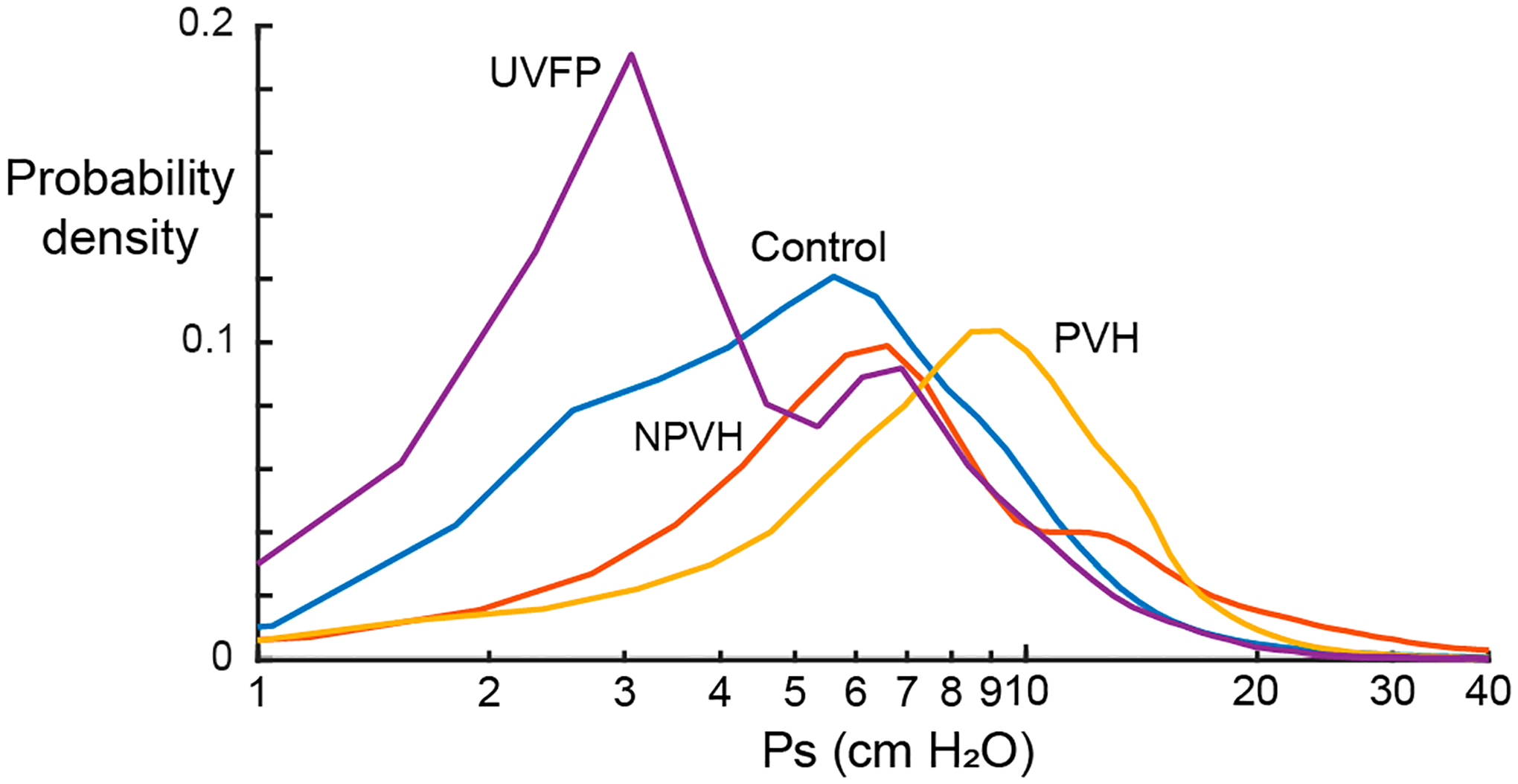
Ambulatory subglottal pressure probability density for each participant group using Ps Estimation Method 3.

Figure 5 displays the probability density functions for ambulatory Ps for each participant group to investigate the ability of real-world monitoring of Ps to discriminate among patient groups and vocally typical speakers. As expected, patients with UVFP displayed the lowest average Ps during daily life, with vocally typical individuals exhibiting the next highest Ps values, followed by patients with NPVH and patients with PVH.

### 3.4. Laboratory versus Ambulatory Distribution of Subglottal Pressure

**Figure 6.**
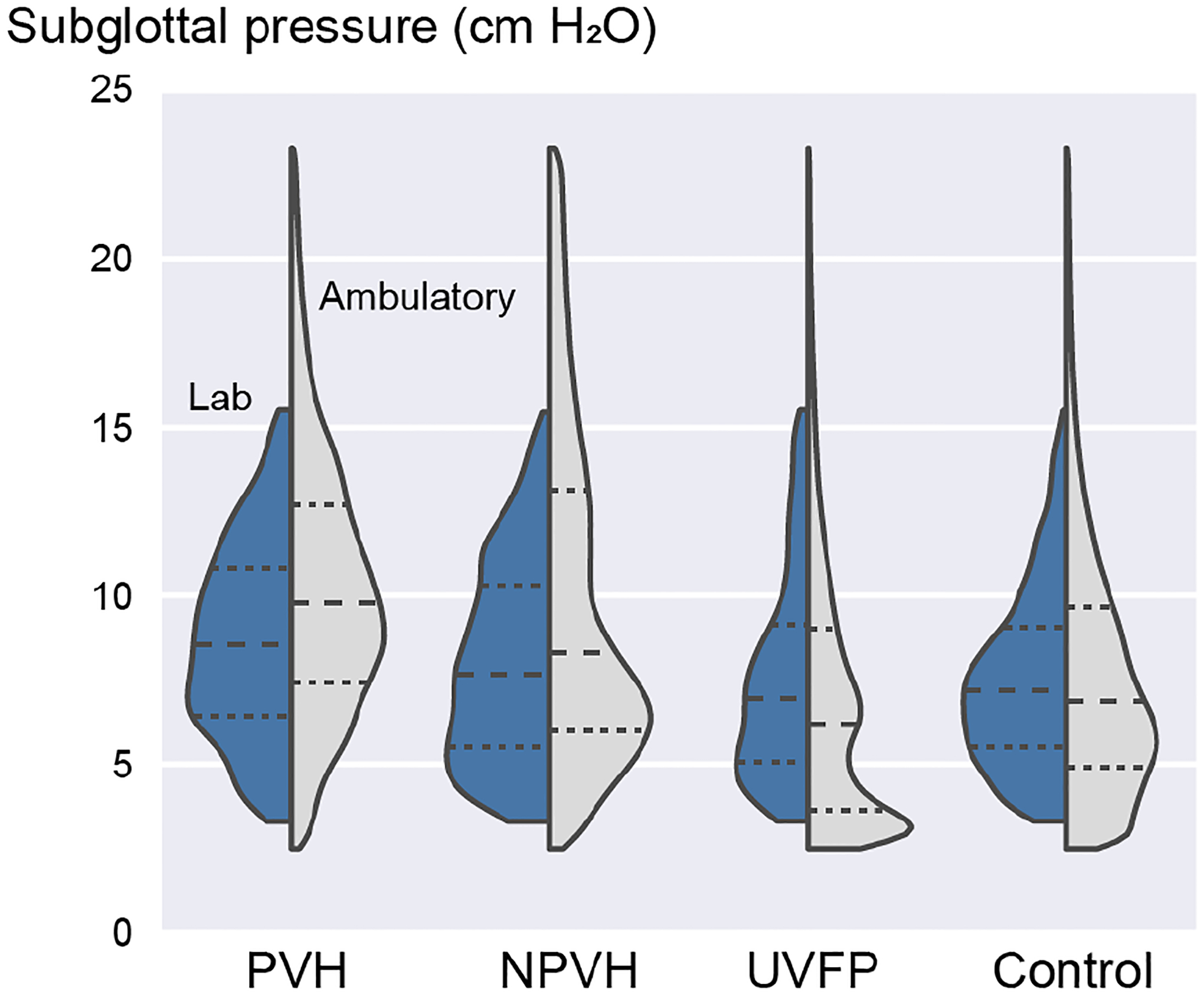
Split-violin plots comparing laboratory (left distribution) and ambulatory (right distribution) estimates of Ps within each participant group using Ps Estimation Method 3.

One may question whether the laboratory recordings elicited vowel segments that spanned the appropriate spectrum of vocal intensity and Ps that individuals exhibit during daily life. A prior study documented the descriptive statistics of SPL, *f*_o_, and Ps (reference Ps from intraoral pressure signals) to demonstrate the range of conditions elicited by the descending-loudness /p/-vowel protocol (Table 2 in [64]). In the laboratory setting, the highest values of Ps produced by participants typically reached 16–18 cm H_2_O. Figure 6 displays the overall Ps distribution for each study participant group when measured in the laboratory setting compared with the estimated Ps distribution in the ambulatory setting (Estimation Method 3). For the vocally typical speaker group, the ambulatory Ps mode was lower than the most frequent Ps elicited in the laboratory setting. Patients with UVFP, expected to exhibit low values of Ps due to glottal incompetence, also exhibited lower average values of Ps in their ambulatory settings relative to what was elicited in the laboratory. In contrast, patients with PVH and NPVH produced higher Ps distributions during their days of monitoring relative to Ps values produced in the laboratory. See Appendix B for split-violin plots displaying laboratory and ambulatory distributions of Ps for each study participant.

## 4. Discussion

The overall goal of the current line of research is a robust method for the non-invasive estimation of Ps during natural speech production that can be applied to laboratory, clinical, and ambulatory monitoring of vocal function. This effort builds upon ongoing work that advances algorithms for analyzing neck-surface vibration monitored using a smartphone platform to enable effective strategies for ambulatory voice monitoring and biofeedback [53,74–80]. A critical missing link in the current set of ambulatory vocal function measures has been the estimation of Ps that would aid in better understanding vocal deficits associated with common voice disorders and would make possible the derivation of additional important vocal metrics (e.g., vocal efficiency [15,36]). Distinguishing among voice modes and vocal pathologies is crucial to obtaining accurate ACC-based estimates of Ps. Subglottal impedance-based inverse filtering (for glottal airflow parameters) [50,77] and vocal function analysis [56] were applied to compute estimates of signal quality and perturbation from the ACC signal. These ACC-based measures were used to help delineate different voice modes in vocally typical speakers and characterize disordered voice production associated with varying degrees of glottal closure, vocal fold stiffness, and vocal fold adductory forces in patients with three types of voice disorders.

From a previous study of ten vocally typical adults in multiple pitch and vowel contexts, the coefficient of determination was significantly higher between ACC RMS and Ps (*r*^*2*^ = 0.68–0.93) than between ACC RMS and acoustic SPL (*r*^*2*^ = 0.46–0.81) [42]. These results suggested that a linear model fit between ACC RMS and Ps could map the ACC signal onto Ps in a time-varying manner. Later work found that the mean (standard deviation) coefficient of determination between ACC RMS and Ps in a group of 26 vocally typical speakers was *r*^2^ = 0.72 (0.14) [63], an average RMSE of 1.7 cm H_2_O [44]. When non-modal phonation was elicited from the speakers with typical voices, the error in estimating Ps using ACC RMS only increased to 2.9 cm H_2_O [63]. The current work confirms that a multiple regression model (Estimation Method 3) performs with the highest Ps estimation accuracy relative to alternative methods by incorporating ACC RMS, *f*_o_, CPP, and glottal airflow measures. Ps estimation error for vocally typical speakers was thus reduced to 1.13 cm H_2_O on average. Ps estimation errors in the patient groups reached minimum values of 1.61, 2.08, and 1.75 cm H_2_O in the PVH, NPVH, and UVFP groups, respectively. Thus, in terms of accuracy, Estimation Method 3 outperformed the three alternative methods compared in this study.

It is worth noting that the most recent Ps estimation method proposed in the literature (Ps Estimation Method 4 [46]) has the advantage of estimating additional measures of phonatory physiology, such as the activation of the thyroarytenoid muscle, cricothyroid muscle, and collision pressure of the vocal folds, but which are out of the scope of the present study. Moreover, Ps Estimation Method 4 was developed as a pilot idea designed for vocally typical female voices producing /pae/-syllable tokens at different loudness conditions with comfortable pitch only; therefore, no males, different pitch conditions, or pathological voices were considered in that study. This is in agreement with the RMSE results for vocally typical participants for Ps Estimation Method 4, which is the lowest error relative to the error in the patient groups. Although the triangular body-cover model has limitations in terms of the *f*_o_ range and offsets of SPL with respect to clinical data that may vary among individuals, the Ps estimation error is comparable to that of the other methods analyzed in this study for the control group. By improving Ps Estimation Method 4 with more simulations for different pathological voice cases, a more robust implementation for estimating Ps for general cases could be obtained, without the necessity of individual models for each speaker (except for individual IBIF models that are still needed to extract aerodynamic measures as input to the neural network).

Although Estimation Method 3 yields the highest Ps estimation accuracy, application of the model requires the computation of several vocal function measures that may each be prone to their own estimation uncertainty. In particular, the IBIF-related glottal airflow measures were only considered valid if they were within physiologically relevant ranges and associated with *f*_o_ values less than 500 Hz. Thus, voiced frames outside of these ranges could not yield glottal airflow measures and, by definition, could not be analyzed using the Ps estimation methods (Estimation Methods 3 and 4) that required these measures as input. This limitation can restrict certain application areas; further work is needed to study scenarios known to exhibit phonation at very high pitch values, including singing voice, infant-directed speech, and pediatric voices. For these scenarios, it would be reasonable to apply Ps Estimation Method 2, which only requires the computation of ACC RMS for input into a single, person-specific regression model. For many study participants, the Ps estimation error for Estimation Method 2 was similar to that exhibited by Estimation Method 3. In addition, the simpler regression model of Estimation Method 2 could be more easily implemented for real-time estimation of Ps as part of a wearable voice monitoring and biofeedback system.

Placed in clinical context, the Ps estimation errors obtained in this study were smaller than known differences in Ps between patients with voice disorders and vocally typical controls. Differences in Ps have been reported to be in the range of 4–5 cm H_2_O for the discrimination of patients with PVH from vocally typical speakers [70]. Furthermore, the strong discriminatory power between patients and controls has been shown to be maintained with ACC-based Ps estimation using Estimation Method 2 (Cohen’s *d* effect sizes up to 1.63) [45]. Reductions in Ps can reach up to 13 cm H_2_O following laryngeal surgery to improve glottal closure for patients with UVFP [71] and laryngeal cancer [24]. Thus, the errors in estimating ACC-based Ps using neck-surface vibration are low enough to use Ps for clinical voice assessment. Calibrating the ACC signal for Ps can also yield an interpretable voice source measure, in contrast to SPL, which is an acoustic measure sensitive to effects of articulation.

In terms of ambulatory voice monitoring, significant progress has been made to characterize patients with PVH who tend to speak with a more restricted pitch range (reduction in *f*_o_ variation), a louder voice more often (the SPL distribution skews toward higher values), and a reduced variability in glottal closure patterns (the distribution of H1–H2 is more restricted) relative to vocally typical individuals [101]. The characterization of changes in Ps promises to provide additional insight into the real-world vocal behavior of individuals with PVH or who are at risk for developing phonotrauma. It is believed that a primary contributing factor to phonotrauma is an increase in vocal fold collision forces during voice production. Since previous work has pointed to a high correlation between vocal fold collision pressure and Ps in certain phonatory scenarios [106,107], ambulatory Ps measures could be used as surrogates for vocal fold collision.

Table 7 documented the average Ps for vocally typical speakers as 8.2 cm H_2_O, with average Ps statistically higher at 11.7 cm H_2_O for patients with PVH. Even more salient is the difference between the trimmed maximum (95th percentile) of ambulatory Ps for the patients with PVH (21.5 cm H_2_O) relative to the control group (15.5 cm H_2_O). This result points to the value in monitoring individuals during their daily activities when they may engage in situations that elicit more extreme voicing—increasing the risk of phonotrauma. Patients with NPVH also exhibit high average (13.9 cm H_2_O) and maximum (25.7 cm H_2_O) values for ambulatory Ps but with higher speaker-to-speaker variability; patients with NPVH are known to exhibit heterogenous voice characteristics, ranging from aphonia to inconsistent vocal stability and vocal fry [103].

The hypothesized ambulatory characteristics are exhibited; e.g., patients with UVFP tend to exhibit higher values of OQ on average (71.4%) than the control group (62.4%) due to glottal incompetence and less abrupt vocal fold closure (Table 8). Furthermore, even more telling is that the patients with UVFP exhibit the minimum ambulatory OQ value of 46.3%, which do not reach the typical minimum value exhibited by the controls during daily life (33.7%). Caution in interpreting specific differences between the patient and control groups is warranted because the control group was not matched to the patient groups in terms of factors that could affect Ps, e.g., occupational vocal demands and sex-specific voice characteristics (male and female speakers both included in the analysis). The current study demonstrated the requisite proof of concept for ambulatory Ps estimation. Future work on larger sample sizes is needed to draw more definitive conclusions regarding ambulatory vocal function and behavior.

Preliminary investigations into determining objective correlates of ambulatory self-ratings of vocal status have yielded limited success using traditional ambulatory measures related to pitch, loudness, and vocal dose [108]. Measures appear to change in both positive and negative directions when increases in vocal effort are reported by speakers [8,108–110]. These traditional measures only assess parameters related to the acoustic output of the voice production system without information from the aerodynamic forces (primarily Ps) needed to generate the voice at the source. There is evidence that the ratio of SPL to Ps (a vocal efficiency-like ratio) can relate to the auditory perception of vocal effort by listeners [111]. Given the evidence supporting the clinical validity of the SPL/Ps ratio [29], there is potential for ambulatory Ps (with acoustic measures of SPL) to accurately reflect the levels of vocal effort being experienced by patients with voice disorders during their activities of daily living.

Differences in Ps distributions can be appreciated between individual laboratory and ambulatory data for most of the study participants (see Appendix B). This result could be attributed to some degree of uncertainty in the estimation of Ps from ambulatory data, either associated with the positioning of the ACC sensor and/or the post processing of IBIF features, which compose the multiple regression model in Ps Estimation Method 3. More control on the process of signal analysis is expected for the laboratory data. For instance, the position of the accelerometer on the subject’s neck sensor might slightly change from laboratory to ambulatory settings, with some variation across participants. The position of the sensor would affect mostly the gain of the ACC signal (and amplitude-based measures of IBIF) [47,50], which is correlated with Ps [42]. It is unlikely that high errors in IBIF estimation have an influence on the Ps distributions, as the ambulatory frames used for analysis were selected so as to have valid IBIF values (Section 2.3.4). In addition, laboratory data include pitch and loudness gestures that might not be typical relative to daily voice-use pitch and loudness. Ambulatory analysis is expected to provide additional information regarding voice use across time, which is not possible to appreciate in the laboratory setting. To minimize errors in the ambulatory setting, it is important to position the accelerometer sensor in approximately the same position to obtain internally consistent voicing measures. Current work is aimed to calibrate the IBIF parameters each day by using an external acoustic microphone, so the participant can easily record the calibration procedure with minimal difficulty and without external assistance [112]. During daily recordings, the participant only must make sure that the sensor is well positioned, and that the phone is recording correctly (any activity that could compromise the sensor position or device should be avoided by pausing or stopping the recording session).

Some studies have questioned the validity of the intraoral pressure method for estimating Ps using /p/-vowel contexts [30], especially in louder conditions [113]. Indeed, critical to the success of this reference approach is that the intraoral pressure waveforms during the plosive are as flat as possible to ensure a valid equilibration of pressures between intraoral and subglottal cavities [114]. The airflow interruption method has been validated using direct measurements of Ps during modal voice production [38,115], but less information is available for individuals with voice disorders. As with most objective clinical measures, caution is suggested when interpreting absolute values of mean Ps obtained using indirect methods, especially for patients with more severe dysphonia for whom the indirect methods have been less studied. Practitioners should incorporate Ps measures as part of the usual comprehensive and multidimensional analysis of vocal function and behavior.

## 5. Conclusions

This study evaluated methods for subglottal pressure estimation based on neck-surface vibration signals. The method exhibiting the lowest error consisted of a person-specific calibration task (repetitions of /p/-vowel syllables at multiple loudness levels) that enabled the training of a multiple regression model that predicted subglottal pressure using a linear combination of vocal function measures. The model was then applied to daylong data collected from vocally typical speakers and patients with phonotraumatic vocal fold lesions, primary muscle tension dysphonia, and unilateral vocal fold paralysis. Ambulatory estimates of subglottal pressure were reported for the first time to obtain a window into the aerodynamics exhibited by individuals during their daily life activities. Future work could investigate the changes in subglottal pressure patterns during the clinical management of an individual’s voice disorder (e.g., following laryngeal surgery or voice therapy sessions), as well as to characterize any sex-based difference during the estimation of subglottal pressure.

## Figures and Tables

**Table 1.** Demographics of the study participants in the three patient groups and the vocally typical control group.

Group	No. Female (Male)	Mean (SD) Age (years)	Age Range (years)
PVH	10 (0)	29 (18)	18–62
NPVH	7 (3)	35 (11)	19–64
UVFP	6 (4)	45 (15)	22–60
Controls	18 (8)	31 (13)	19–50

PVH = phonotraumatic vocal hyperfunction; NPVH = nonphonotraumatic vocal hyperfunction; UVFP = unilateral vocal fold paralysis; Controls = vocally typical control group.

**Table 2.** Accelerometer-based vocal function measures input into Ps Estimation Methods 3 and 4. See Figure 3 for an illustration of the waveform and spectra parameterization.

Measure	Units	Description
RMS	cm/s^2^	Root-mean-square signal magnitude
*f*_o_	Hz	Fundamental frequency
CPP	dB	Cepstral peak prominence
ACFL	mL/s	Peak-to-peak amplitude of the glottal airflow waveform
MFDR	L/s^2^	Maximum flow declination rate: Negative peak of the first derivative of the glottal airflow waveform
OQ	%	Open quotient: Ratio of the open time of the glottal airflow waveform to the corresponding cycle period (*t*_O_/*t*_C_)
SQ	%	Speed quotient: Ratio of the opening time of the glottal airflow waveform to the closing time (100 × *t*_op_/*t*_cp_)
H1–H2	dB	Difference between the log-magnitude of the first two harmonics of the glottal airflow waveform
HRF	dB	Harmonic richness factor: Ratio of the sum of the first eight harmonic log-magnitudes to the first harmonic magnitude of the glottal airflow waveform
NAQ	a.u.	Normalized amplitude quotient: Ratio of ACFL to MFDR (ACFL/MFDR) divided by the glottal period (*t*O + *t*C) of the glottal airflow waveform

**Table 3.** Description of accelerometer-based features and voice-activity detection (VAD) range criteria for each feature computed on in-field ambulatory voice data to determine whether a 50-ms frame was considered voiced or unvoiced.

Feature	Units	VAD Criteria	Description
Sound pressure level @ 15 cm	dB SPL	45–130	Acceleration amplitude mapped to acoustic sound pressure level [57]
Fundamental frequency	Hz	70–1000	Reciprocal of first non-zero peak location in the normalized autocorrelation function [53]
Autocorrelation peak amplitude	a.u.	0.60–1	Amplitude of first non-zero peak in the normalized autocorrelation function [77,98]
Subharmonic peak	a.u.	0.25–1	Amplitude of a secondary peak, if it exists, located between the zero-lag and the autocorrelation peak in the normalized autocorrelation function [77,98]
Low-to-high spectral power ratio	dB	22–50	Difference between spectral power below and above 2000 Hz [77]

**Table 4.** Error of the four subglottal pressure (Ps) estimation methods for each patient group and vocally typical group in terms of root-mean-square error (units of cm H_2_O) with respect to reference Ps values obtained using the indirect intraoral equilibration method. The mean and standard deviation (SD) of the error are listed. Reported also for the patient groups are the mean (SD) of the auditory-perceptual rating of overall severity (higher values on the 0–100 scale indicate higher dysphonia).

Group	Method 1	Method 2	Method 3	Method 4	Overall Severity
PVH	4.10 (3.06)	2.10 (1.04)	1.61 (0.60)	3.18 (1.73)	22.4 (18.0)
NPVH	3.76 (1.62)	2.08 (0.90)	2.08 (0.60)	3.51 (2.07)	25.3 (29.5)
UVFP	4.74 (2.44)	2.06 (0.71)	1.75 (0.67)	4.86 (2.66)	54.5 (27.4)
Control	2.96 (1.42)	1.51 (0.48)	1.13 (0.37)	2.89 (0.82)	N/A

**Table 5.** Results of the two-way analysis of variance on the root-mean-square error in subglottal pressure (Ps) estimation to determine the main effects of and interactions between the participant group and estimation method.

Effect	*df*	$\eta_{p}^{2}$	*F*	*p*
Participant Group	3	0.188	8.32	<0.0001
Ps Estimation Method	3	0.750	33.25	<0.0001
Participant Group × Ps Estimation Method	9	0.062	0.91	0.516

**Table 6.** For each participant group, the average inclusion frequency (count and percentage) is reported for each vocal function measure that was input into Ps Estimation Method 3 (multiple regression model).

Group	RMS	CPP	*F*_o_	ACFL	MFDR	OQ	NAQ	HRF	H1-H2
PVH (*n* = 10)	9 (90%)	6 (60%)	9 (90%)	4 (40%)	7 (70%)	5 (50%)	5 (50%)	2 (20%)	4 (40%)
NPVH (*n* = 10)	8 (80%)	7 (70%)	6 (60%)	5 (50%)	5 (50%)	5 (50%)	3 (30%)	4 (40%)	3 (30%)
UVFP (*n* = 10)	10 (100%)	2 (20%)	6 (60%)	4 (40%)	7 (70%)	2 (20%)	4 (40%)	4 (40%)	4 (40%)
Control (*n* = 26)	25 (96%)	16 (62%)	18 (69%)	11 (42%)	13 (50%)	10 (38%)	10 (38%)	13 (50%)	14 (54%)
Average (%)	92%	53%	70%	43%	60%	40%	40%	38%	41%

Speed quotient not included since it was not included in any of the models.

**Table 7.** Univariate statistics of daylong ambulatory estimates of subglottal pressure (Ps) using Estimation Method 3 (multiple regression model) for each participant group, along with other vocal function measures computed from the accelerometer signal: sound pressure level (SPL), cepstral peak prominence (CPP), and the difference between the first two harmonic magnitudes (H1–H2). Phonation time is reported in minutes and seconds (mm:ss) and percentage units. Group-based *f*_o_ statistics are not reported due to the known differences in *f*_o_ for male and female speakers.

Ambulatory Statistic	PVH	NPVH	UVFP	Control
Monitoring duration (hh:mm:ss)	11:27:43 (04:28:38)	10:24:52 (02:18:50)	10:21:54 (03:36:12)	10:51:05 (02:49:34)
Phonation time				
Cumulative (mm:ss)	48:45 (32:39)	52:56 (33:22)	28:39 (23:22)	44:43 (28:14)
Normalized (%)	7.1 (4.7)	8.5 (5.3)	4.6 (3.8)	6.9 (4.3)
Ps (cm H_2_O)				
Mean	11.7 (2.8)	13.9 (8.1)	8.2 (3.3)	8.2 (3.6)
Mode	9.2 (4.3)	8.1 (4.0)	5.8 (4.4)	6.1 (2.7)
Standard deviation	5.3 (1.9)	6.4 (5.5)	2.7 (2.0)	3.9 (2.7)
Skewness	1.957 (0.908)	1.613 (0.540)	2.474 (0.813)	2.309 (1.017)
Minimum *	5.8 (1.5)	6.3 (2.5)	5.6 (3.1)	4.3 (1.3)
Maximum *	21.5 (5.7)	25.7 (18.0)	13.3 (6.2)	15.5 (9.1)
SPL (dB SPL @ 15 cm)				
Mean	87.0 (8.0)	82.7 (11.5)	85.8 (8.4)	86.0 (8.6)
Standard deviation	7.7 (2.3)	7.2 (2.4)	7.0 (3.0)	7.2 (2.6)
Skewness	−0.448 (0.417)	−0.459 (0.279)	−0.073 (0.476)	−0.187 (0.357)
Minimum *	73.5 (4.2)	73.1 (7.2)	70.6 (11.5)	64.8 (8.1)
Maximum *	97.2 (4.8)	98.0 (9.4)	94.0 (12.8)	93.7 (9.7)
CPP (dB)				
Mean	22.0 (1.3)	18.8 (2.0)	21.7 (1.7)	18.7 (1.9)
Standard deviation	4.2 (0.4)	3.1 (0.7)	3.9 (0.8)	3.1 (0.7)
Skewness	−0.233 (0.219)	−0.212 (0.209)	−0.037 (0.180)	−0.115 (0.281)
Minimum *	14.9 (0.6)	14.7 (0.7)	13.5 (0.8)	14.6 (0.8)
Maximum *	28.6 (1.1)	28.3 (1.5)	23.7 (2.9)	28.4 (1.7)
H1−H2 (dB)				
Mean	4.5 (2.1)	8.3 (3.3)	5.7 (4.0)	8.3 (3.3)
Standard deviation	7.6 (1.1)	5.8 (1.1)	6.8 (2.0)	5.9 (1.0)
Skewness	0.673 (0.433)	0.669 (0.452)	0.202 (0.394)	0.552 (0.366)
Minimum *	−3.8 (1.6)	−5.8 (3.3)	−0.6 (3.7)	−3.7 (3.0)
Maximum *	15.6 (2.7)	18.5 (2.7)	18.4 (2.9)	18.1 (3.6)

* Minimum and maximum are trimmed estimators reporting the 5th percentile and 95th percentile, respectively.

**Table 8.** Univariate statistics of daylong ambulatory estimates of glottal airflow measures for each participant group: peak-to-peak glottal airflow (ACFL), maximum flow declination rate (MFDR), open quotient (OQ), speed quotient (SQ), the difference between the first two harmonic magnitudes (H1–H2), harmonic richness factor (HRF), and normalized amplitude quotient (NAQ).

Ambulatory Statistic	PVH	NPVH	UVFP	Control
ACFL (mL/s)				
Mean	337.7 (213.3)	482.6 (409.4)	131.4 (131.0)	195.6 (173.0)
Standard deviation	267.5 (178.3)	390.8 (325.6)	109.3 (105.6)	158.7 (142.2)
Skewness	2.493 (1.653)	2.282 (0.652)	2.805 (1.570)	2.680 (0.935)
Minimum	48.4 (33.0)	81.1 (88.0)	23.5 (26.5)	35.2 (34.5)
Maximum *	831.9 (555.9)	1260.7 (1089.7)	323.6 (321.5)	481.2 (430.7)
MFDR (L/s^2^)				
Mean	529.2 (344.1)	737.9 (624.5)	166.5 (175.2)	296.6 (287.1)
Standard deviation	481.9 (324.9)	659.7 (557.1)	169.7 (175.2)	277.5 (254.7)
Skewness	2.765 (1.779)	2.478 (0.853)	3.292 (1.161)	3.021 (0.899)
Minimum *	49.8 (34.6)	91.2 (97.0)	21.8 (24.8)	35.5 (39.0)
Maximum *	1424.1 (978.9)	2057.5 (1797.4)	468.4 (487.9)	792.7 (738.6)
OQ (%)				
Mean	58.3 (6.9)	57.2 (7.3)	71.4 (10.1)	62.4 (9.5)
Standard deviation	19.2 (2.5)	19.3 (1.4)	15.3 (3.8)	19.3 (3.2)
Skewness	0.554 (0.591)	0.503 (0.337)	−0.301 (0.806)	0.109 (0.576)
Minimum *	33.0 (4.2)	31.8 (7.0)	46.3 (12.7)	33.7 (8.1)
Maximum *	92.6 (4.2)	92.1 (2.5)	94.7 (2.7)	93.7 (2.4)
SQ (%)				
Mean	148.0 (10.9)	143.6 (11.2)	137.6 (15.9)	147.7 (14.9)
Standard deviation	69.9 (19.5)	63.2 (21.6)	61.1 (22.8)	73.4 (18.8)
Skewness	2.145 (0.520)	2.079 (0.629)	1.573 (0.457)	1.857 (0.516)
Minimum *	64.1 (23.1)	63.6 (23.7)	60.2 (14.4)	55.1 (14.5)
Maximum *	290.9 (66.1)	267.5 (65.4)	251.1 (69.8)	290.4 (65.2)
H1–H2 (dB)				
Mean	4.2 (4.5)	2.9 (3.4)	8.9 (6.9)	6.1 (3.9)
Standard deviation	10.8 (2.3)	13.3 (5.8)	9.3 (2.6)	10.2 (2.4)
Skewness	0.069 (0.825)	0.676 (0.417)	0.078 (0.765)	0.280 (0.595)
Minimum *	−12.3 (4.2)	−17.7 (13.9)	−4.2 (8.0)	−8.8 (4.3)
Maximum *	20.4 (4.4)	27.4 (13.6)	24.3 (4.7)	22.0 (5.2)
HRF (dB)				
Mean	−2.5 (1.3)	−3.5 (2.9)	−7.6 (3.6)	−3.9 (2.4)
Standard deviation	6.7 (1.6)	8.9 (4.2)	6.8 (1.9)	7.6 (1.9)
Skewness	−3.556 (0.979)	−2.803 (1.085)	−1.390 (1.556)	−2.709 (1.164)
Minimum *	−15.7 (5.2)	−23.7 (16.4)	−20.2 (4.4)	−18.2 (5.7)
Maximum *	2.2 (0.6)	2.3 (1.2)	−0.2 (2.6)	2.1 (0.9)
NAQ				
Mean	0.169 (0.013)	0.173 (0.031)	0.212 (0.025)	0.177 (0.024)
Standard deviation	0.056 (0.007)	0.059 (0.009)	0.057 (0.008)	0.063 (0.009)
Skewness	1.917 (0.546)	1.593 (0.451)	1.201 (0.629)	1.387 (0.517)
Minimum *	0.106 (0.012)	0.106 (0.023)	0.136 (0.023)	0.102 (0.019)
Maximum *	0.285 (0.023)	0.291 (0.043)	0.316 (0.020)	0.302 (0.028)

* Minimum and maximum are trimmed estimators reporting the 5th percentile and 95th percentile, respectively.

**Figure A1.**
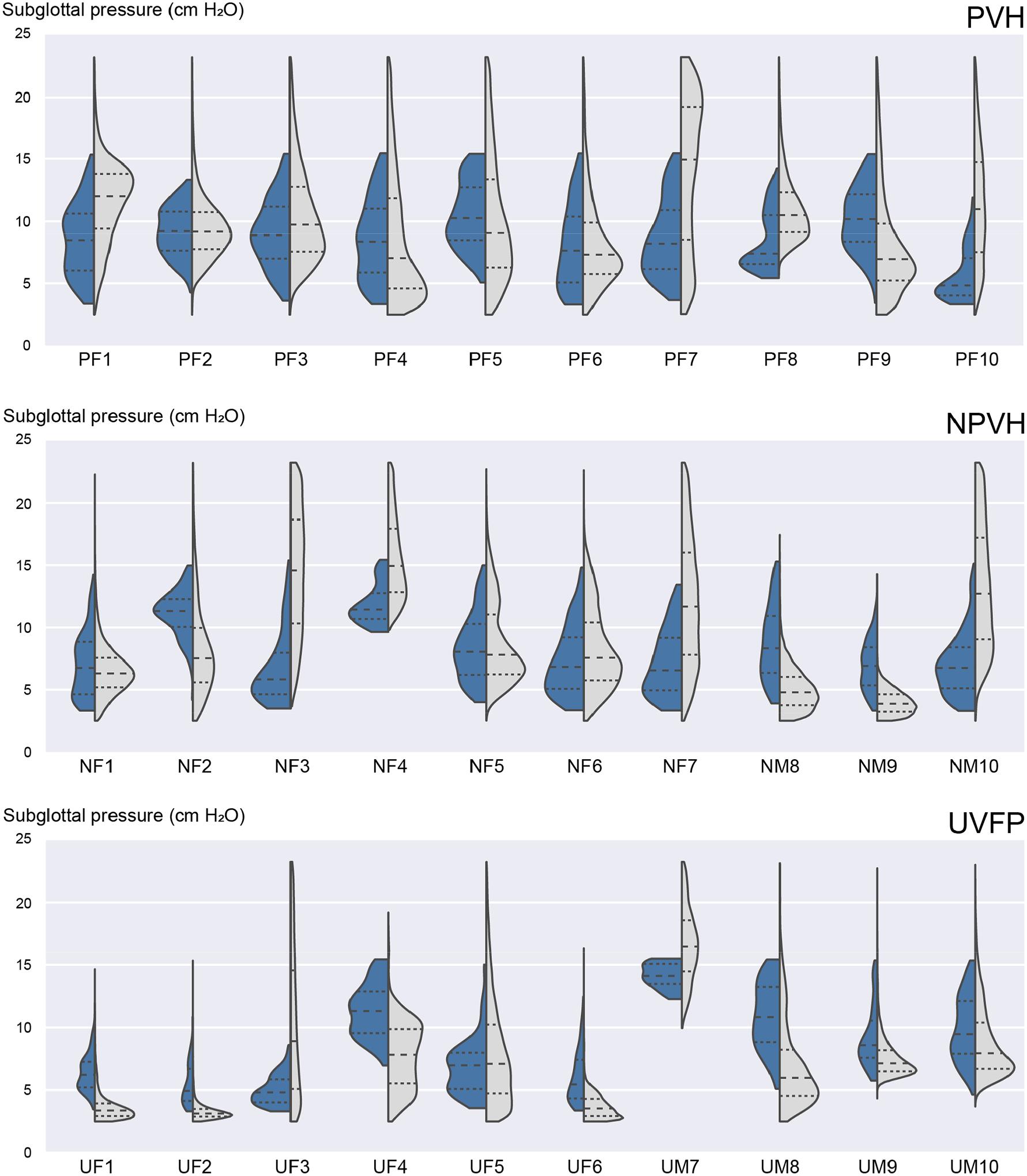
Split-violin plots comparing laboratory (left distribution) and ambulatory (right distribution) estimates of Ps for each patient.

**Figure A2.**
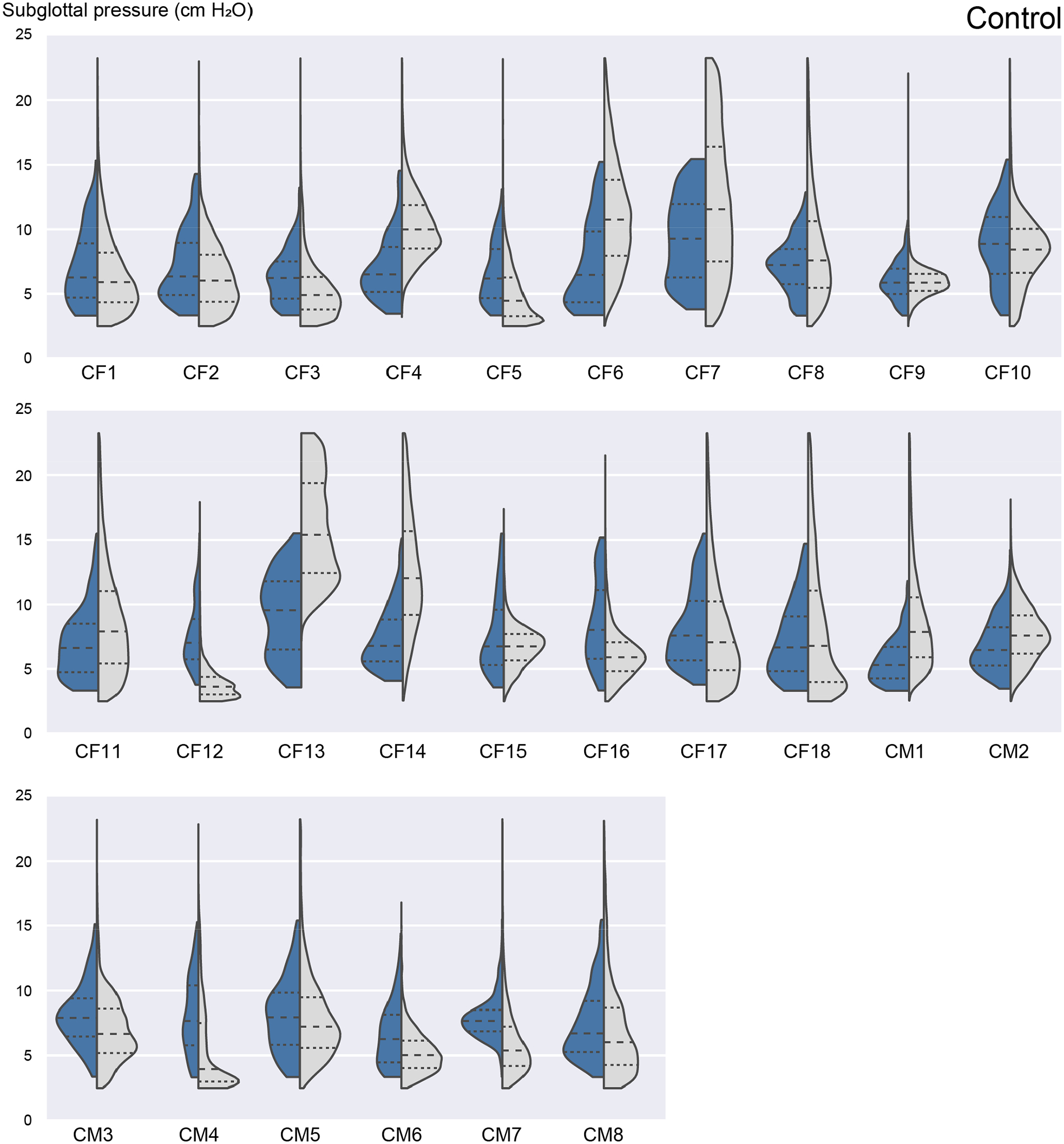
Split-violin plots comparing laboratory (left distribution) and ambulatory (right distribution) estimates of Ps for each vocally typical participant.

## Data Availability

Mass General Brigham and Mass General are not allowed to give access to data without the Principal Investigator (PI) for the human studies protocol first submitting a protocol amendment to request permission to share the data with a specific collaborator on a case-by-case basis. This policy is based on very strict rules dealing with the protection of patient data and information. Anyone wishing to request access to the data must first contact Ms. Sarah DeRosa, Program Coordinator for Research and Clinical Speech-Language Pathology, Center for Laryngeal Surgery and Voice Rehabilitation, Massachusetts General Hospital: sederosa@partners.org.

## Appendix A

Table A1 (patients) and Table A2 (vocally typical individuals) list the errors of the four Ps estimation methods for each study participant in terms of root-mean-square error with respect to reference Ps values measured using the indirect intraoral equilibration method. For patients, the auditory-perceptual ratings of overall severity are also reported (higher values on the 0–100 scale indicate higher dysphonia).

**Table A1.** Error of the four subglottal pressure (Ps) estimation methods for each patient in terms of root-mean-square error (units of cm H_2_O) with respect to reference Ps values measured using the indirect intraoral equilibration method. Reported also are the auditory-perceptual ratings of overall severity.

ID *	Method 1	Method 2	Method 3	Method 4	Overall Severity
PF1	2.45	2.03	1.65	2.61	3
PF2	2.73	1.48	1.32	1.89	14
PF3	2.42	1.59	1.02	2.60	21
PF4	5.81	2.11	1.56	4.12	11
PF5	1.82	1.78	1.03	1.75	50
PF6	11.51	1.98	1.84	3.78	0
PF7	3.35	1.63	1.42	2.81	18
PF8	2.69	2.44	2.18	2.61	37
PF9	6.43	4.85	2.96	7.58	51
PF10	1.74	1.11	1.09	2.00	19
NF1	4.78	1.92	1.45	4.77	23
NF2	3.58	2.54	2.31	3.94	62
NF3	1.37	1.06	1.05	2.29	9
NF4	7.67	3.96	3.96	8.52	93
NF5	3.82	1.61	0.97	1.97	7
NF6	3.05	2.61	2.23	2.51	26
NF7	3.12	0.84	0.74	1.77	4
NM8	3.61	2.56	2.30	4.21	7
NM9	2.90	2.03	1.19	1.71	4
NM10	3.66	1.69	1.49	3.37	18
UF1	5.20	1.36	1.31	3.89	57
UF2	5.87	1.23	1.10	4.34	77
UF3	2.02	1.98	1.75	2.44	56
UF4	9.82	2.43	2.08	8.49	73
UF5	3.17	2.57	2.42	2.34	48
UF6	2.29	1.86	0.96	1.61	95
UM7	6.49	2.11	1.70	9.84	19
UM8	3.24	3.15	2.88	6.02	77
UM9	6.28	1.04	0.98	5.36	10
UM10	3.06	2.90	2.36	4.25	33

* First two characters of ID indicate patient type (P = PVH; N = NPVH; U = UVFP) and sex (M = male; F = female).

**Table A2.** Error of the four Ps estimation methods for each vocally typical participant in terms of root-mean-square error (units of cm H_2_O) with respect to reference Ps values measured using the indirect intraoral equilibration method.

ID *	Method 1	Method 2	Method 3	Method 4
CF1	2.61	1.29	0.73	2.41
CF2	1.69	1.04	0.78	2.55
CF3	2.43	1.34	0.76	4.30
CF4	2.78	1.47	1.31	3.22
CF5	1.44	1.09	0.57	2.90
CF6	1.39	0.98	0.87	1.96
CF7	4.16	2.47	2.12	3.81
CF8	2.18	1.47	1.28	2.54
CF9	4.68	1.17	1.04	3.81
CF10	2.03	1.48	1.23	2.57
CF11	4.59	1.68	1.39	3.26
CF12	4.68	1.56	1.29	2.75
CF13	2.34	1.90	1.23	2.38
CF14	2.35	0.82	0.73	2.52
CF15	1.88	1.05	0.81	2.22
CF16	4.08	2.36	1.74	5.44
CF17	5.29	1.92	1.29	1.94
CF18	1.79	1.28	0.84	2.24
CM1	4.54	0.85	0.69	2.06
CM2	1.21	1.29	0.85	1.88
CM3	2.26	2.33	1.58	3.51
CM4	6.58	2.31	1.06	3.19
CM5	2.31	1.82	1.33	2.93
CM6	3.64	0.97	0.87	2.83
CM7	2.49	1.63	1.50	2.55
CM8	1.58	1.61	1.41	3.41

* First two characters of ID indicate participant type (C = vocally typical control) and sex (M = male; F = female).

## Appendix B

Figure A1 (patients) and Figure A2 (vocally typical individuals) display split-violin plots comparing laboratory and daylong ambulatory estimates of Ps for each study participant.
